# Mathematical analysis and molecular descriptors of two novel metal–organic models with chemical applications

**DOI:** 10.1038/s41598-023-32347-4

**Published:** 2023-03-31

**Authors:** Shahid Zaman, Mehwish Jalani, Asad Ullah, Wakeel Ahmad, Ghulamullah Saeedi

**Affiliations:** 1 Department of Mathematics, University of Sialkot, Sialkot, 51310 Pakistan; 2 Department of Mathematical Sciences, Karakoram International University Gilgit, Gilgit, 15100 Pakistan; 3 Department of Mathematics, Polytechnical University of Kabul, Kabul, Afghanistan

**Keywords:** Cheminformatics, Computational chemistry, Structure prediction, Applied mathematics

## Abstract

Metal–Organic Networks (MONs) are made by chemical molecules that contain metal ions and organic ligands. A crystalline porous solid called Metal–Organic Networks (MONs) is made up of a $3D$ metal network of ions held in place by a multidentate ligand. (MONs) can be used for gas storage, purification drug delivery, gas separation, catalysis, and sensing applications. There is enormous potential for effective integration and research of MONs in diverse applications. Molecular descriptors are arithmetic measures that reveal a chemical substance's physical and chemical characteristics in its foundational network in a natural relationship. They demonstrate an important role in theoretical and ecological chemistry, and in the field of medicine. In this research, we calculated various recently discovered molecular descriptors viz. the modified version of second zagreb index, harmonic index, reciprocal randic index, modified version of forgotten topological index, redefined first zagreb topological index, redefined second zagreb topological index and redefined third zagreb topological index for two separate metal–organic networks. The numerical and graphical comparative analysis of these considered molecular descriptors are also performed.

## Introduction

Every heavenly body is a combination of several constituents that significantly contributes to the composition of the earth. The three-element hydrogen, oxygen and nitrogen are the most significant ones on the planet^1,2^. Massively used compounds made by chemically like metal–organic network (MON) are thus made up of alloy ions/metallic ions and organic linkers. With the aid of the hydrothermal method, new MONs composed of zinc considered metal ions and benzene $\mathrm{1,3}$-dicarboxylic acid as the organic linker^3^. Biography of MONs is their superficial alteration and as well as their particle control six division^4^. Devices with luminous properties could be created using $Zn$-related MONs, are chemical sensors^1^. In reality,${Zn}^{+2}$ an astringent, anti-dandruff, antibacterial, and anti-inflammatory autogenous simple powerless noxious conversion metal cation, is frequently worked in homoeopaths as a scarring catalyst and face ointment^5^. Additionally, the production processes of MONs related to zinc have lately been documented, and as well their toxicity, biological uses, and biocompatibility^6^. In modern chemistry, graph theory offers essential tools that depict chemical compounds' heats of formation, evaporation, flash points, temperatures, pressures, densities, and partition coefficients^7^. Zinc oxide is a white powder that is insoluble in water. Nanostructure of Zinc oxide $(Zno)$ can be synthesized into a range of different morphologies. A variety of skin conditions can be treated with zinc oxide. In marine environments, zinc silicate coating can provide long-term protection of steel and is used in rapid coating work for over fifty years. Zinc oxide is also used in toothpastes to prevent plaque. These metals also help the human body, it is present in the red blood cells and causes several reactions related to carbon dioxide metabolism. Zinc silicate networks are economical because they are relatively thin coating.

The zinc oxide and zinc silicate exhibit physicochemical characteristics including grafting active groups^8^, incorporating appropriate active material^9^, ion exchange^10^, creating composites using various materials^11^, modifying organic ligands, photosynthetic ligands, and improving the selectivity, sensitivity, and response times of biosensors.Yap et al.^12^ and Lin et al.^13^ presented the most current developments in precursors for a variety of nanostructures and MON-related applications, including lithium ion batteries, super capacitors, photocatalysis, electrocatalysis, and catalysts for the manufacture of fine chemicals. The field of modern chemistry can benefit from using graph theory to represent the physical and chemical characteristics of chemical compounds, such as their heats of formation and evaporation, flash points, melting points, boiling points, temperatures, pressures, densities, retention times in chromatography, tensions, and partition coefficients^14,15^. In order to investigate many characteristics of chemical compounds (such as the boiling point of paraffin), Wiener first developed the distance-based topological index (TI) in 1947. Gutman and Trinajsic's highly regarded first-degree-based TI was developed to test the chemical plausibility of the total π-electron energy of the chemical compounds (alternant hydrocar-bons).

The Zagreb type indices contributed tremendously in the various fields which can be seen in^16–21^. The details on other degree based molecular descriptors and structures are given in^22–27^. In 2021, the authors of^28^ computed the connection-based Zagreb indices such as first Zagreb connection index (ZCI), second ZCI, modified first ZCI, modified second ZCI, modified third ZCI, and modified fourth ZCI. We extended those results for other degree based topological indices as Modified version of second Zagreb index ${M}_{2}\left(G\right)$, Harmonic index $H\left(G\right)$^29^, Reciprocal Randic index $RR\left(G\right)$, the Modified version of Forgotten topological index ${F}_{N}^{*}$, the Redefined First Zagreb topological index ${R}_{e}Z{G}_{1}(G)$^30^, the Redefined Second Zagreb topological index ${R}_{e}Z{G}_{2}\left(G\right)$^31^ as well as the Redefined third Zagreb topological index ${R}_{e}Z{G}_{3}(G)$^32^ for MONs, namely Zinc oxide $\left(ZNOX\left(n\right)\right)$ and zinc silicate $\left(ZNSL\left(n\right)\right)$ as regards to the expanding layers,$n\ge 3$. Our outcomes fascinate not only mathematician but also of theoretical chemists. The results of this study can be used to examine numerical quantities and guide future research into the physical properties of molecules. As a consequence, it is a beneficial procedure to eliminate costly and time-consuming laboratory studies. The findings of this research depict that ${R}_{e}Z{G}_{3}\left(G\right)$ achieved higher values than other classical Zagreb indices, which may have better correlation with the thirteen physicochemical characteristics of octane isomers.

## Main results

Here, we initially present some significant definitions of the degree based molecular descriptors which will be useful to obtain the main results. In the whole study, we denote the adjacent vertices by $p$ and $q$, i.e. $pq\in {E}_{G}.$

### Definition 1

The molecular descriptor ${M}_{2}\left(G\right)$ denotes modified version of second zagreb index that is described as^33^,

$${M}_{2}\left(G\right)={\sum }_{pq \in E\left(G\right)} \frac{1}{{d}_{G}\left(p\right)\times {d}_{G}\left(q\right)}$$

### Definition 2

The molecular descriptor $H\left(G\right)$ denotes harmonic index which is defined in^34^ as,

$$H\left(G\right)={\sum }_{pq \in E\left(G\right)} \frac{2}{{d}_{G}\left(p\right)+{d}_{G}\left(q\right)}$$

### Definition 3

The molecular descriptor $RR\left(G\right)$ denotes reciprocal randic index which is explained as^35^,

$$RR\left(G\right)={\sum }_{pq \in E\left(G\right)}\sqrt{{d}_{G}\left(p\right)\times {d}_{G}\left(q\right)}$$

### Definition 4

The molecular descriptor ${F}_{N}^{*}$ denotes modified version of forgotten topological index, that is described as^36^,

$${F}_{N}^{*}\left(G\right)={\sum }_{pq\in E\left(G\right) }{[d}_{G}{\left(p\right)}^{2} +{d}_{G}{\left(q\right)}^{2}]$$

### Definition 5

The molecular descriptor ${R}_{e}Z{G}_{1}$ denotes redefined first zagreb topological index, which is defined by^36^,

$${R}_{e}Z{G}_{1}(G)={\sum }_{pq\in E\left(G\right)}\frac{{d}_{G}\left(p\right)+{d}_{G}\left(q\right)}{{d}_{G}\left(p\right)\times {d}_{G}\left(q\right)}$$

### Definition 6

The molecular descriptor ${R}_{e}Z{G}_{2}(G)$ denotes the redefined second zagreb topological index, that is described as^31^,

$${R}_{e}Z{G}_{2}(G)={\sum }_{pq \in E\left(G\right)}\frac{{d}_{G}\left(p\right)\times {d}_{G}\left(q\right)}{{d}_{G}\left(p\right)+{d}_{G}\left(q\right)}$$

### Definition 7

The molecular descriptor ${R}_{e}Z{G}_{3}(G)$ denotes redefined third zagreb topological index, which is explained by^31^,

$${R}_{e}Z{G}_{3}\left(G\right)={\sum }_{pq \in E\left(G\right)}{d}_{G}\left(p\right)\times {d}_{G}\left(q\right)\left[{d}_{G}\left(p\right)+{d}_{G}\left(q\right)\right]$$

### Theorem 1

**Figure 1:**
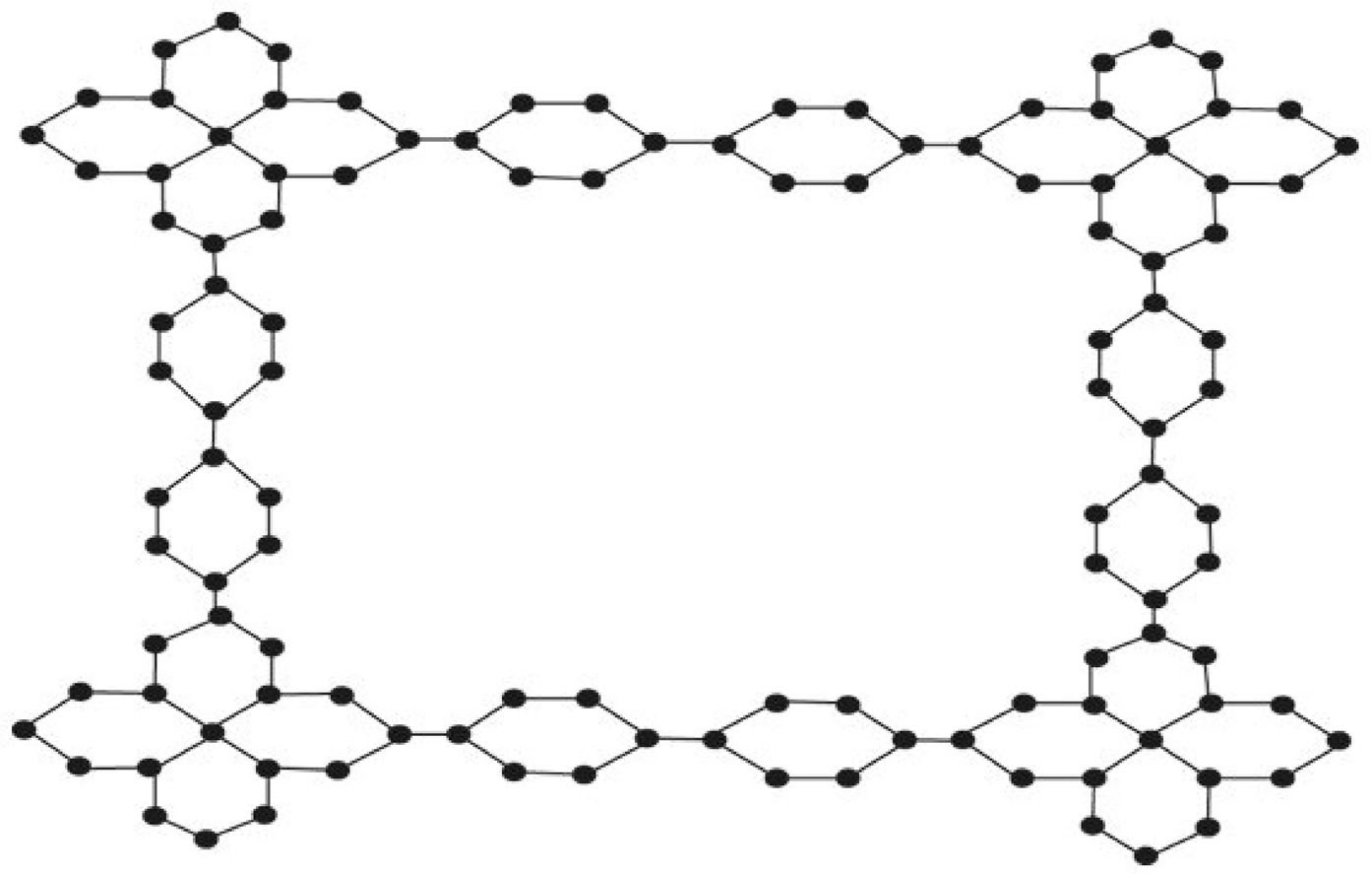
Zinc oxide network (ZNOX (n) $\cong$ H), where $n=3$.

Let $H\cong ZNOX\left(n\right)$ is a zinc oxide network as depicted in Fig. 1, then

$${M}_{2}\left(G\right)=11.928n+9.7$$

### Proof

Based on the Definition 1 and Table 1, we have

**Table 1:** Edge partition of $ZNOX$ in relation to the degrees.

${E}_{p,q}^{d}$	${E}_{\mathrm{2,2}}^{d}$	${E}_{\mathrm{2,3}}^{d}$	${E}_{\mathrm{3,3}}^{d}$	${E}_{\mathrm{3,4}}^{d}$
|$${E}_{p,q}^{d}|$$	$$6n+16$$	$$52n+28$$	$$9n+3$$	$$8n+8$$

$${M}_{2}\left(G\right)={\sum }_{pq \in E\left(G\right)} \frac{1}{{d}_{G}\left(p\right)\times {d}_{G}\left(q\right)} ={\sum }_{pq \in {E}_{\mathrm{2,2}}} \frac{1}{{d}_{G}\left(p\right)\times {d}_{G}\left(q\right)} +{\sum }_{pq \in {E}_{\mathrm{2,3}}} \frac{1}{{d}_{G}\left(p\right)\times {d}_{G}\left(q\right)} +{\sum }_{pq \in {E}_{\mathrm{3,3}}} \frac{1}{{d}_{G}\left(p\right)\times {d}_{G}\left(q\right)} +{\sum }_{pq \in {E}_{\mathrm{3,4}}} \frac{1}{{d}_{G}\left(p\right)\times {d}_{G}\left(q\right)} =\left|{E}_{\mathrm{2,2}}\right|\left(\frac{1}{2\times 2}\right)+\left|{E}_{\mathrm{2,3}}\right|\left(\frac{1}{2\times 3}\right)+ \left|{E}_{\mathrm{3,3}}\right|\left(\frac{1}{3\times 3}\right)+\left|{E}_{\mathrm{3,4}}\right|\left(\frac{1}{3\times 4}\right) =\frac{\left(6n+16\right)}{4} +\frac{\left(52n+28\right) }{6}+\frac{ \left(9n+3\right)}{9}+\frac{\left(8n+8\right)}{12} {M}_{2}\left(G\right)=11.928n+9.7$$

### Theorem 2

Let $H\cong ZNOX\left(n\right)$ be a zinc oxide network as shown in Fig. 1, then

$$H\left(G\right)=29.01n+22.43$$

### Proof

Based on the Definition 2 and Table 1, one has

$$\begin{gathered} H\left( G \right) = \mathop \sum \limits_{pq \in E\left( G \right)} \frac{2}{{d_{G} \left( p \right) + d_{G} \left( q \right)}} \hfill \\ = \left| {E_{2,2} } \right|\left( {\frac{2}{2 + 2}} \right) + \left| {E_{2,3} } \right|\left( {\frac{2}{2 + 3}} \right) + \left| {E_{3,3} } \right|\left( {\frac{2}{3 + 3}} \right) + \left| {E_{3,4} } \right|\left( {\frac{2}{3 + 4}} \right) \hfill \\ = 3n + 8 + 20.8n + 11.2 + 2.97n + 0.99 + 2.24n + 2.24 \hfill \\ H\left( G \right) = 29.01n + 22.43. \hfill \\ \end{gathered}$$

### Theorem 3

Let $H\cong ZNOX\left(n\right)$ be a zinc oxide network as shown in Fig. 1, then

$$\mathrm{RR}\left(\mathrm{G}\right)=193.56\mathrm{n}+137$$

### Proof

Based on the Definition 3 and Table 1, one has

$$\begin{gathered} RR\left( G \right) = \mathop \sum \limits_{pq \in E\left( G \right)} \sqrt {d_{G} \left( p \right) \times d_{G} \left( q \right)} \hfill \\ = \sqrt {2 \times 2} \left| {E_{2,2} } \right| + \sqrt {2 \times 3} \left| {E_{2,3} } \right| + \sqrt {3 \times 3} \left| {E_{3,3} } \right| + \sqrt {3 \times 4} \left| {E_{3,4} } \right| \hfill \\ = \sqrt 4 \times \left( {6n + 16} \right) + \sqrt 6 \times \left( {52n + 28} \right) + \sqrt 9 \times \left( {9n + 3} \right) + \sqrt {12} \times \left( {8n + 8} \right) \hfill \\ {\text{RR}}\left( {\text{G}} \right) = 193.56{\text{n}} + 137 \hfill \\ \end{gathered}$$

### Theorem 4

Let $H\cong ZNOX\left(n\right)$ be a zinc oxide network as shown in Fig. 1, then

$${\mathrm{R}}_{\mathrm{e}}{\mathrm{ZG}}_{1}(\mathrm{G})=59.97\mathrm{n}+46$$

### Proof

Based on the Definition 5 and Table 1, we have

$$\begin{gathered} R_{e} ZG_{1} \left( G \right) = \mathop \sum \limits_{pq \in E\left( G \right)} \frac{{d_{G} \left( p \right) + d_{G} \left( q \right)}}{{d_{G} \left( p \right) \times d_{G} \left( q \right)}} \hfill \\ = \frac{2 + 2}{{2 \times 2}}\left| {E_{2,2} } \right| + \frac{2 + 3}{{2 \times 3}}\left| {E_{2,3} } \right| + \frac{3 + 3}{{3 \times 3}}\left| {E_{3,3} } \right| + \frac{3 + 4}{{3 \times 4}}\left| {E_{3,4} } \right| \hfill \\ \end{gathered}$$

$${\mathrm{R}}_{\mathrm{e}}{\mathrm{ZG}}_{1}(\mathrm{G})=59.97n+46$$

The following corollary is a direct consequent of Theorem 4.

### Corollary 5

Let $H\cong ZNOX\left(n\right)$ be a zinc oxide network as depicted in Fig. 1, then

$${R}_{e}Z{G}_{2}(G)=95.612n+67.812$$

### Theorem 6

Let $H\cong ZNOX\left(n\right)$ be a zinc oxide network as shown in Fig. 1, then

$${R}_{e}Z{G}_{3}\left(G\right)=2814n+1930$$

### Proof

Based on the Definition 7 and Table 1, we have

$$\begin{gathered} R_{e} ZG_{3} \left( G \right) = \mathop \sum \limits_{pq \in E\left( G \right)} d_{G} \left( p \right) \times d_{G} \left( q \right)\left[ { d_{G} \left( p \right) + d_{G} \left( q \right)} \right] \hfill \\ = \left( 2 \right)\left( 2 \right)\left[ {2 + 2} \right]\left| {{\text{E}}_{2,2} } \right| + \left( 2 \right)\left( 3 \right)\left[ {2 + 3} \right]\left| {{\text{E}}_{2,3} } \right| + \left( 3 \right)\left( 3 \right)\left[ {3 + 3} \right]\left| {{\text{E}}_{3,3} } \right| + \left( 3 \right)\left( 4 \right)\left[ {3 + 4} \right]\left| {{\text{E}}_{3,4} } \right| \hfill \\ = 4\left( 4 \right)\left( {6{\text{n}} + 16} \right) + 6\left( 5 \right)\left( {52{\text{n}} + 28} \right) + 9\left( 6 \right)\left( {9{\text{n}} + 3} \right) + \left( {12} \right)7\left( {8{\text{n}} + 8} \right) \hfill \\ \end{gathered}$$

$${R}_{e}Z{G}_{3}\left(G\right)=2814n+1930$$

### Theorem 7

Let $H\cong ZNOX\left(n\right)$ be a zinc oxide network as depicted in Fig. 1, then

$${F}_{N}^{*}\left(G\right)=1086n+746$$

### Proof

Based on the Definition 4 and Table 1, one has

$${F}_{N}^{*}\left(G\right)={\sum }_{pq\in E\left(G\right) }{[d}_{G}{\left(p\right)}^{2} +{d}_{G}{\left(q\right)}^{2}] ={\sum }_{pq\in E\left(\mathrm{2,2}\right) }{[d}_{G}{\left(p\right)}^{2} +{d}_{G}{\left(q\right)}^{2}]+{\sum }_{pq\in E\left(\mathrm{2,3}\right) }{[d}_{G}{\left(p\right)}^{2} +{d}_{G}{\left(q\right)}^{2}]+{\sum }_{pq\in E\left(\mathrm{3,3}\right) }{[d}_{G}{\left(p\right)}^{2} +{d}_{G}{\left(q\right)}^{2}]+{\sum }_{pq\in E\left(\mathrm{3,4}\right) }{[d}_{G}{\left(p\right)}^{2} +{d}_{G}{\left(q\right)}^{2}]$$

$${F}_{N}^{*}\left(G\right)=1086n+746$$

### Theorem 8

**Figure 2:**
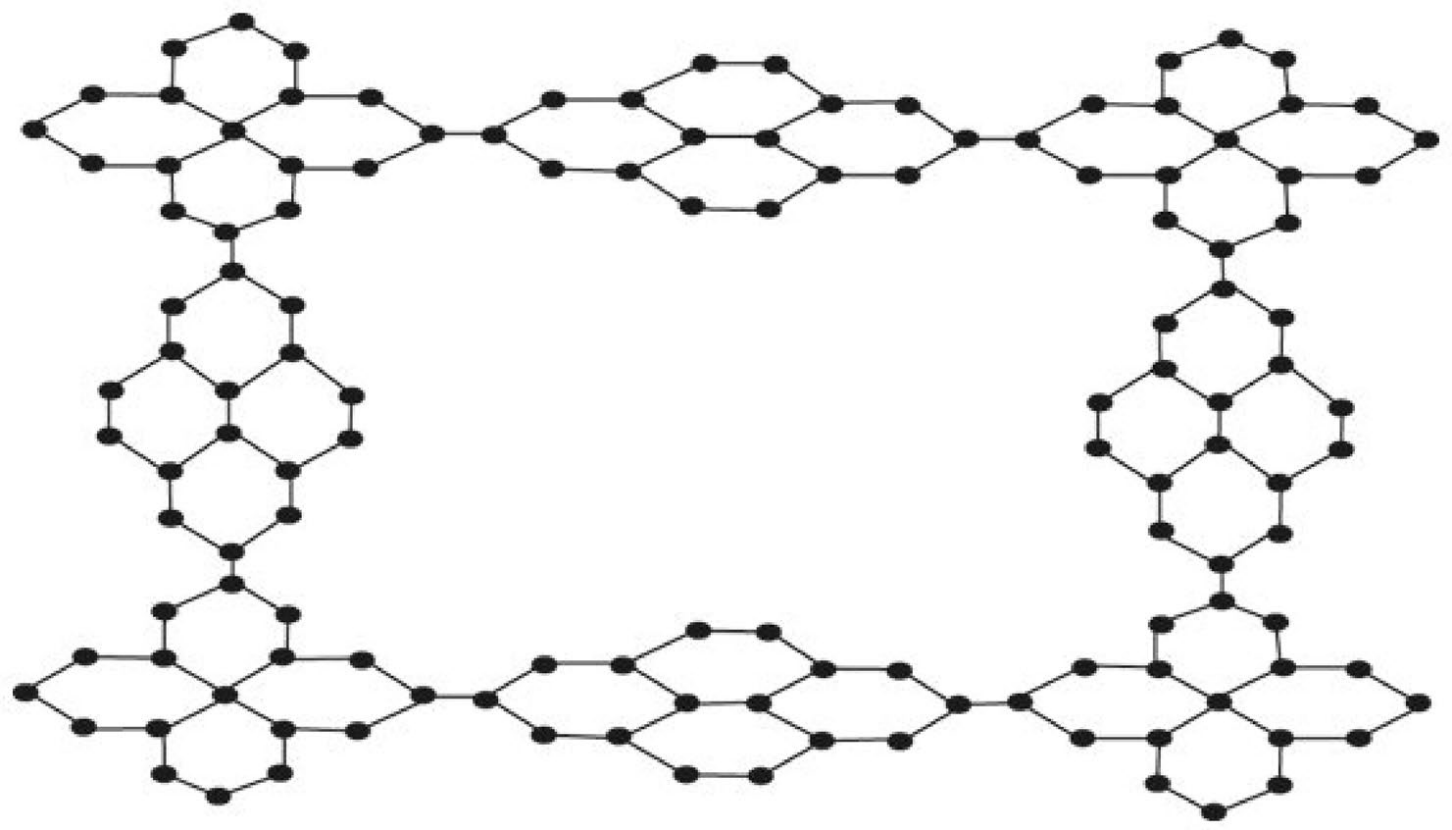
Zinc silicate network (ZNSL (n) $\cong$ K), where $n=3$.

Suppose $K\cong ZNSL\left(n\right)$ be a zinc silicate network as depicted by Fig. 2, then

$${M}_{2}\left(G\right)=16.17n+10.28$$

### Proof

Based on the Definition 1 and Table 2, we have

$$\begin{gathered} M_{2} \left( G \right) = \mathop \sum \limits_{pq \in E\left( G \right)} \frac{1}{{d_{G} \left( p \right) \times d_{G} \left( q \right)}} \hfill \\ = \mathop \sum \limits_{{pq \in E_{2,2} }} \frac{1}{{d_{G} \left( p \right) \times d_{G} \left( q \right)}} + \mathop \sum \limits_{{pq \in E_{2,3} }} \frac{1}{{d_{G} \left( p \right) \times d_{G} \left( q \right)}} + \mathop \sum \limits_{{pq \in E_{3,3} }} \frac{1}{{d_{G} \left( p \right) \times d_{G} \left( q \right)}} + \mathop \sum \limits_{{pq \in E_{3,4} }} \frac{1}{{d_{G} \left( p \right) \times d_{G} \left( q \right)}} \hfill \\ = \left| {E_{2,2} } \right|\left( {\frac{1}{2 \times 2}} \right) + \left| {E_{2,3} } \right|\left( {\frac{1}{2 \times 3}} \right) + \left| {E_{3,3} } \right|\left( {\frac{1}{3 \times 3}} \right) + \left| {E_{3,4} } \right|\left( {\frac{1}{3 \times 4}} \right) \hfill \\ \end{gathered}$$

**Table 2:** Edge partition of $ZNSL$ in relation to the degrees.

${E}_{p,q}^{d}$	${E}_{\mathrm{2,2}}^{d}$	${E}_{\mathrm{2,3}}^{d}$	${E}_{\mathrm{3,3}}^{d}$	${E}_{\mathrm{3,4}}^{d}$
|$${E}_{p,q}^{d}|$$	$$10n+14$$	$$64n+32$$	$$21n+7$$	$$8n+8$$

$${M}_{2}\left(G\right)=16.17n+10.28$$

### Theorem 9.

Suppose $K\cong ZNSL\left(n\right)$ be a zinc silicate network as shown by Fig. 2, then

$$H\left(G\right)=39.88n+24.41$$

### Proof

Based on the Definition 2 and Table 2, we have

$$\begin{gathered} H\left( G \right) = \mathop \sum \limits_{pq \in E\left( G \right)} \frac{2}{{d_{G} \left( p \right) + d_{G} \left( q \right)}} \hfill \\ = \mathop \sum \limits_{{pq \in E\left( {2,2} \right)}} \frac{2}{{d_{G} \left( p \right) + d_{G} \left( q \right)}} + \mathop \sum \limits_{{pq \in E\left( {2,3} \right)}} \frac{2}{{d_{G} \left( p \right) + d_{G} \left( q \right)}} + \mathop \sum \limits_{{pq \in E\left( {3,3} \right)}} \frac{2}{{d_{G} \left( p \right) + d_{G} \left( q \right)}} + \mathop \sum \limits_{{pq \in E\left( {3,4} \right)}} \frac{2}{{d_{G} \left( p \right) + d_{G} \left( q \right)}} \hfill \\ = \left| {E_{2,2} } \right|\left( {\frac{2}{2 + 2}} \right) + \left| {E_{2,3} } \right|\left( {\frac{2}{2 + 3}} \right) + \left| {E_{3,3} } \right|\left( {\frac{2}{3 + 3}} \right) + \left| {E_{3,4} } \right|\left( {\frac{2}{3 + 4}} \right) \hfill \\ \end{gathered}$$

$$H\left(G\right)=39.88n+24.41$$

### Theorem 10

Suppose $K\cong ZNSL\left(n\right)$ be a zinc silicate network as shown by Fig. 2, then

$$RR\left(G\right)=266.84n+154.76$$

### Proof

Based on the Definition 3 and Table 2, one has

$$\begin{gathered} {\text{RR}}\left( {\text{G}} \right) = \mathop \sum \limits_{pq \in E\left( G \right)} \sqrt {d_{G} \left( p \right) \times d_{G} \left( q \right)} \hfill \\ = \sqrt {2 \times 2} \left| {E_{2,2} } \right| + \sqrt {2 \times 3} \left| {E_{2,3} } \right| + \sqrt {3 \times 3} \left| {E_{3,3} } \right| + \sqrt {3 \times 4} \left| {E_{3,4} } \right| \hfill \\ = \sqrt 4 \left( {10n + 14} \right) + \sqrt 6 \left( {64n + 32} \right) + \sqrt 9 \left( {21n + 7} \right) + \sqrt {12} \left( {8n + 8} \right) \hfill \\ \end{gathered}$$

$$\mathrm{RR}\left(\mathrm{G}\right)=266.84\mathrm{n}+154.76$$

### Theorem 11

Suppose $K\cong ZNSL\left(n\right)$ be a zinc silicate network as depicted by Fig. 2,

$${R}_{e}Z{G}_{1}(G)=82n+50.01$$

### Proof

Based on the Definition 5 and Table 2, one has

$$\begin{gathered} {\text{R}}_{{\text{e}}} {\text{ZG}}_{1} \left( {\text{G}} \right) = \mathop \sum \limits_{pq \in E\left( G \right)} \frac{{d_{G} \left( p \right) + d_{G} \left( q \right)}}{{d_{G} \left( p \right) \times d_{G} \left( q \right)}} \hfill \\ = \frac{2 + 2}{{2 \times 2}}\left| {E_{2,2} } \right| + \frac{2 + 3}{{2 \times 3}}\left| {E_{2,3} } \right| + \frac{3 + 3}{{3 \times 3}}\left| {E_{3,3} } \right| + \frac{3 + 4}{{3 \times 4}}\left| {E_{3,4} } \right| \hfill \\ \end{gathered}$$

$${\mathrm{R}}_{\mathrm{e}}{\mathrm{ZG}}_{1}(\mathrm{G})=82n+50.01$$

The following corollary is a direct consequent of Theorem 11.

### Corollary 12

Suppose $K\cong ZNSL\left(n\right)$ be a zinc silicate network as shown by Fig. 2, then

$${R}_{e}Z{G}_{2}(G)=132.01\mathrm{n}+76.61$$

### Theorem 13

Suppose $K\cong ZNSL\left(n\right)$ be a zinc silicate network as shown by Fig. 2, then

$${R}_{e}Z{G}_{3}\left(G\right)=3886n+2234$$

### Proof

Based on the Definition 7 and Table 2, one has

$$\begin{gathered} R_{e} ZG_{3} \left( G \right) = \mathop \sum \limits_{pq \in E\left( G \right)} d_{G} \left( p \right) \times d_{G} \left( q \right)\left[ { d_{G} \left( p \right) + d_{G} \left( q \right)} \right] \hfill \\ = \left( 2 \right)\left( 2 \right)\left[ {2 + 2} \right]\left| {{\text{E}}_{2,2} } \right| + \left( 2 \right)\left( 3 \right)\left[ {2 + 3} \right]\left| {{\text{E}}_{2,3} } \right| + \left( 3 \right)\left( 3 \right)\left[ {3 + 3} \right]\left| {{\text{E}}_{3,3} } \right| + \left( 3 \right)\left( 4 \right)\left[ {3 + 4} \right]\left| {{\text{E}}_{3,4} } \right| \hfill \\ = 4\left( 4 \right)\left( {10{\text{n}} + 14} \right) + 6\left( 5 \right)\left( {64{\text{n}} + 32} \right) + 9\left( 6 \right)\left( {21{\text{n}} + 7} \right) + \left( {12} \right)7\left( {8{\text{n}} + 8} \right) \hfill \\ \end{gathered}$$

$${R}_{e}Z{G}_{3}\left(G\right)=3886n+2234$$

### Theorem 14

Suppose $K\cong ZNSL\left(n\right)$ be a zinc silicate network as shown by Fig. 2, then

$${\mathrm{F}}_{\mathrm{N}}^{*}\left(\mathrm{G}\right)=1490\mathrm{n}+854$$

### Proof

Considering Table 2 and Definition 4, we can write

$$\begin{gathered} F_{N}^{*} ( G ) = \mathop \sum \limits_{pq \in E( G ) } [d_{G} ( p )^{2} + d_{G} ( q )^{2} ] \hfill \\ = \mathop \sum \limits_{{pq \in E( {2,2} ) }} [d_{G} ( p )^{2} + d_{G} ( q )^{2} ] { + \mathop \sum \limits_{{pq \in E( {2,3} ) }} [d_{G} ( p )^{2} + d_{G} ( q )^{2} } ] \hfill \\ + \mathop \sum \limits_{{pq \in E( {3,3} ) }} [d_{G} ( p )^{2} + d_{G} ( q )^{2} ] { + \mathop \sum \limits_{{pq \in E( {3,4} ) }} [d_{G} ( p )^{2} + d_{G} ( q )^{2} } ] \hfill \\ = [ 8 ]( {10n + 14} ) + [ {13} ]( {64n + 32} ) + [ {18} ]( {21n + 7} ) + [ {25} ]( {8n + 8} ) \hfill \\ \end{gathered}$$

$${F}_{N}^{*}\left(G\right)=1490n+85$$

## Graphical interpretations

**Figure 3:**
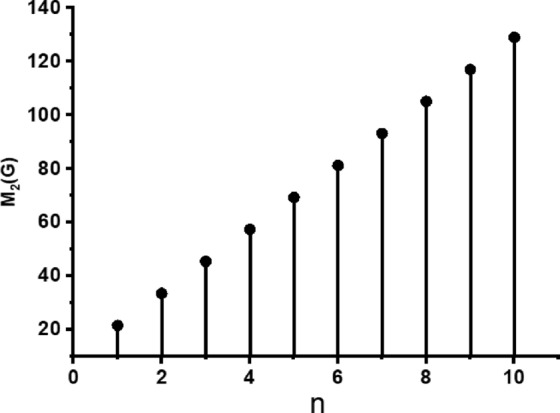
The comparison of $n$ and ${M}_{2}\left(G\right)$.

**Figure 4:**
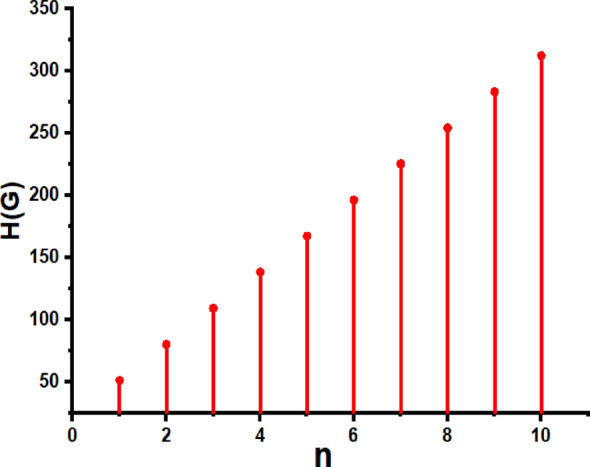
The comparison of $n$ and .$H\left(G\right)$

**Figure 5:**
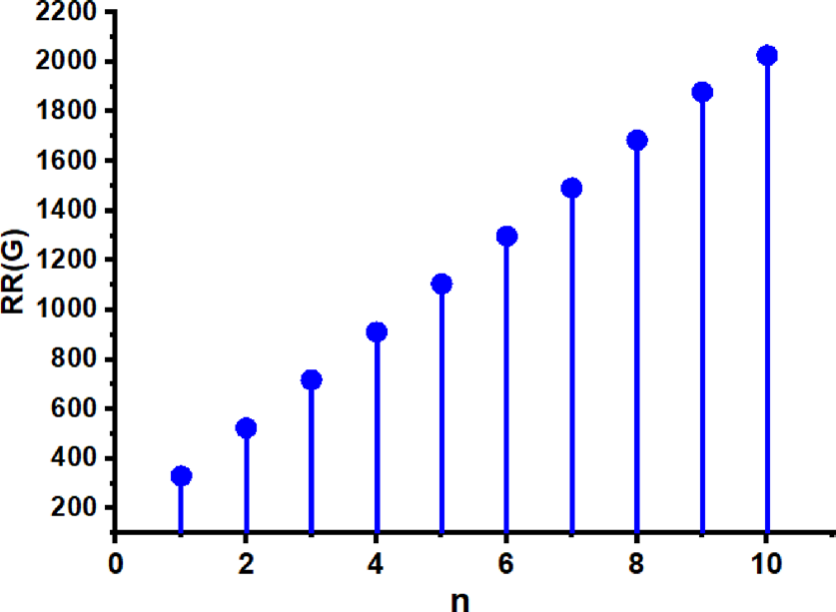
The comparison of $n$ and .$RR\left(G\right)$

**Figure 6:**
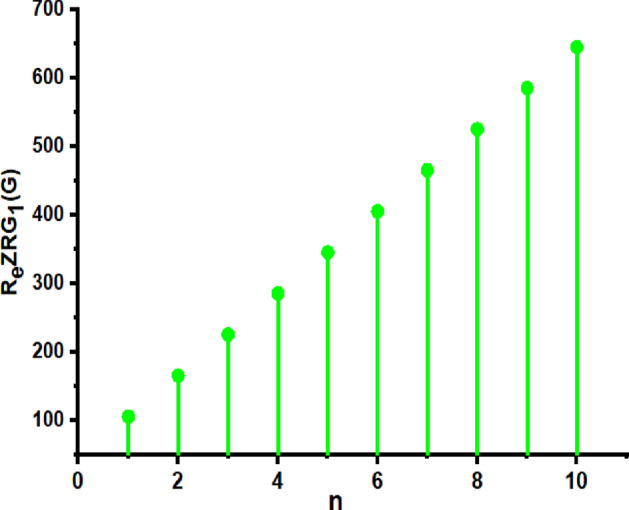
The comparison of $n$ and .${R}_{e}Z{RG}_{1}\left(G\right)$

**Figure 7:**
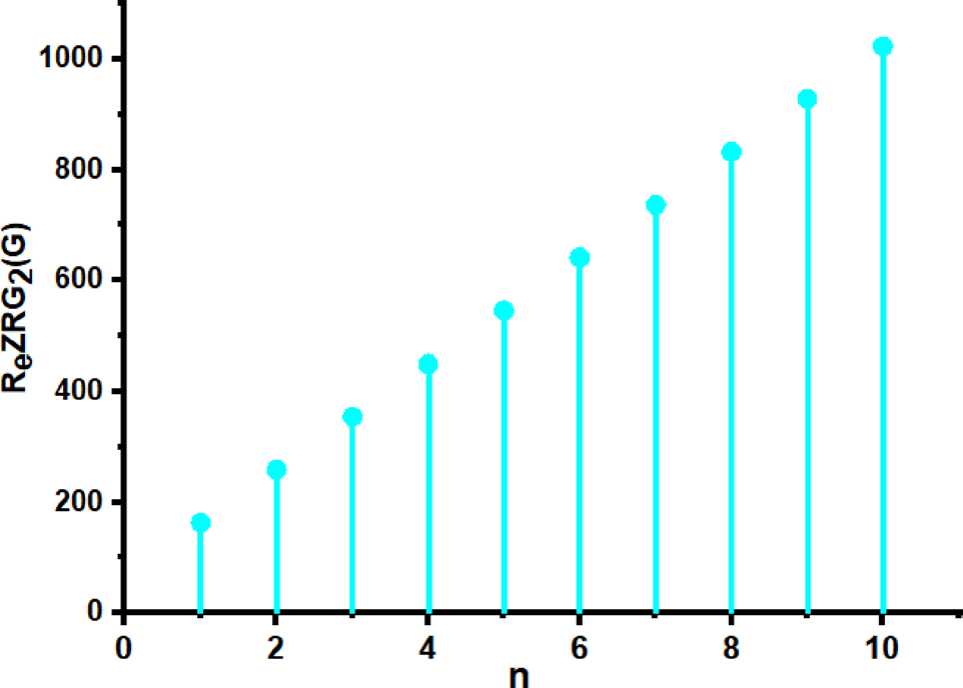
The comparison of $n$ and .${R}_{e}Z{RG}_{2}\left(G\right)$

**Figure 8:**
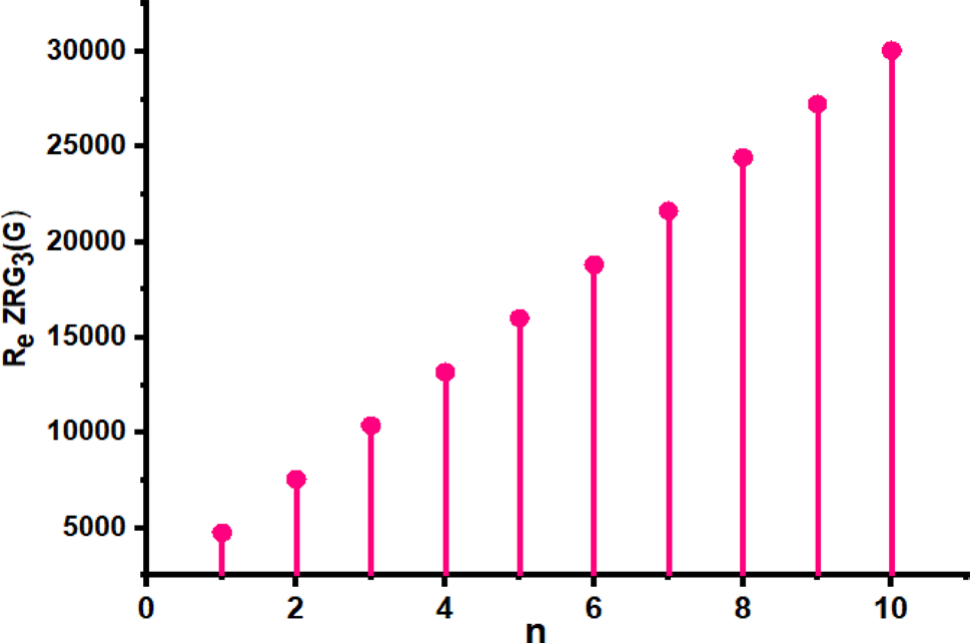
The comparison of $n$ and .${R}_{e}Z{RG}_{3}\left(G\right)$

**Figure 9:**
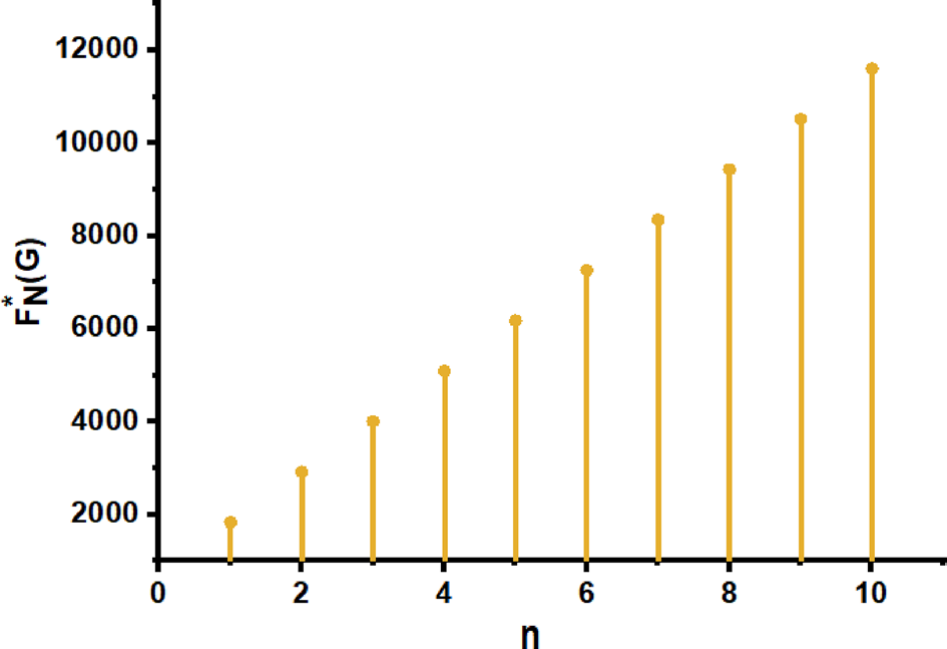
The comparison of $n$ and ${F}_{N}^{*}\left(G\right)$.

**Figure 10:**
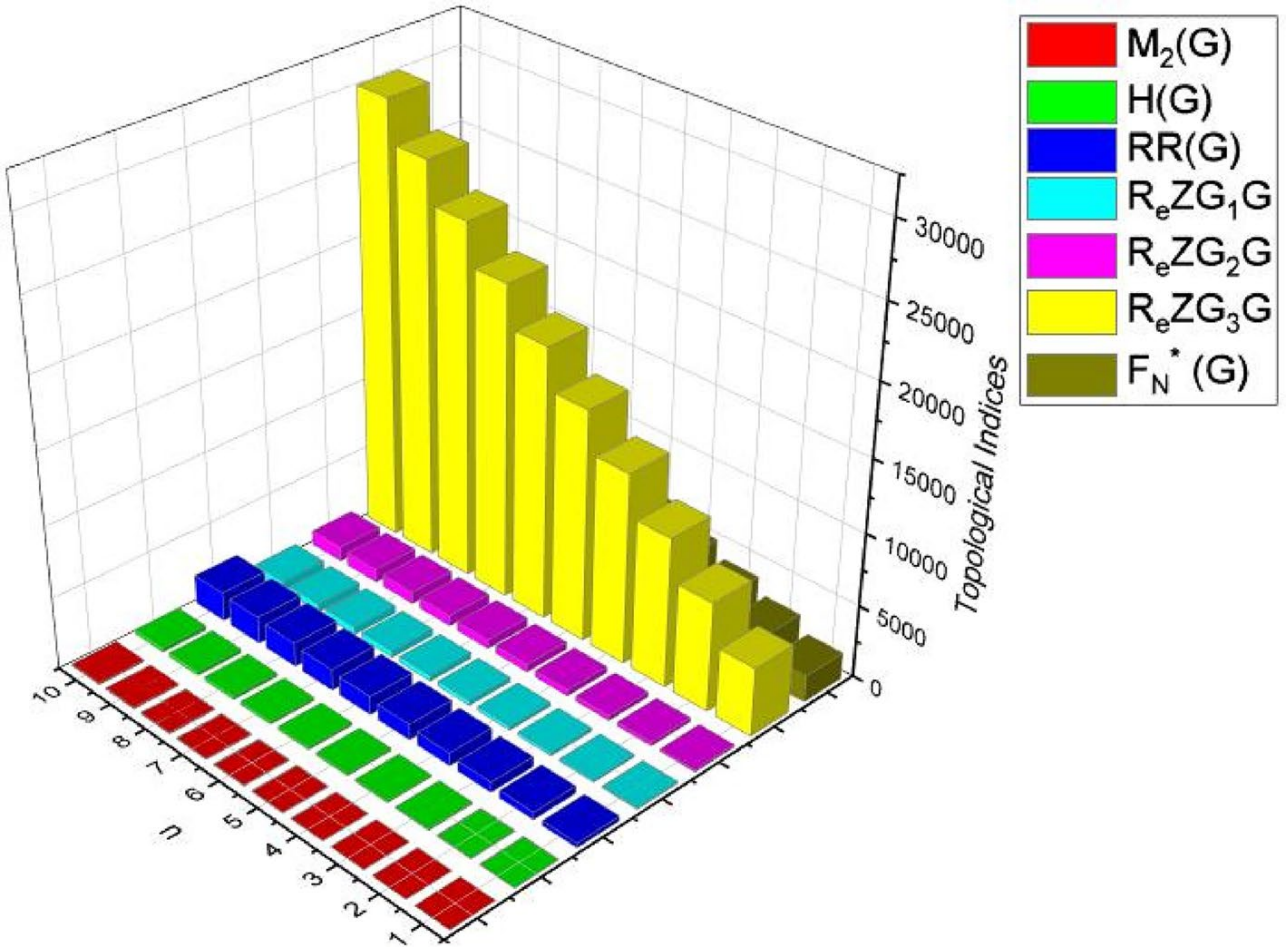
Comparison of topological indices for various values of $n$ in $\mathrm{ZNOX}$.

In this part, we worked out all indices numerically and presented the results in the table below. From Figs. 3, 4, 5, 6, 7, 8, 9 and Table 3, it is easy to see a positive relationship between *n* and the considered topological indices. As we increase *n*, the topological indices also increase. The comparative graphs of ${\mathrm{M}}_{2}\left(\mathrm{G}\right)$, $\mathrm{H}\left(\mathrm{G}\right)$, $\mathrm{RR}\left(\mathrm{G}\right)$, ${\mathrm{R}}_{\mathrm{e}}{\mathrm{ZG}}_{1}(\mathrm{G})$, ${\mathrm{R}}_{\mathrm{e}}{\mathrm{ZG}}_{2}(\mathrm{G})$, ${\mathrm{R}}_{\mathrm{e}}{\mathrm{ZG}}_{3}(\mathrm{G})$ and ${\mathrm{F}}_{\mathrm{N}}^{*}\left(\mathrm{G}\right)$ indices of $\mathrm{ZNOX}$ for various values are presented in Fig. 10. Thus, Fig. 10 describe that all indices for $\mathrm{ZNOX}$ increase for increasing value of *n*. The increasing rate of ${R}_{e}Z{G}_{3}(G)$ is higher than that of other topological indices. This depict that, ${R}_{e}Z{G}_{3}\left(G\right)$ achieved higher values than other classical Zagreb indices, which may have better correlation with the thirteen physicochemical characteristics of octane isomers.

**Table 3:** Comparison of ${\mathrm{M}}_{2}\left(\mathrm{G}\right)$, $\mathrm{H}\left(\mathrm{G}\right)$, $\mathrm{RR}\left(\mathrm{G}\right)$, ${\mathrm{R}}_{\mathrm{e}}{\mathrm{ZG}}_{1}(\mathrm{G})$, ${\mathrm{R}}_{\mathrm{e}}{\mathrm{ZG}}_{2}(\mathrm{G})$, ${\mathrm{R}}_{\mathrm{e}}{\mathrm{ZG}}_{3}(\mathrm{G})$ and ${\mathrm{F}}_{\mathrm{N}}^{*}\left(\mathrm{G}\right)$ for $\mathrm{ZNOX}$.

[$n$]	${M}_{2}\left(G\right)$	$H\left(G\right)$	$RR\left(G\right)$	${R}_{e}Z{G}_{1}(G)$	${R}_{e}Z{G}_{2}(G)$	${R}_{e}Z{G}_{3}(G)$	${F}_{N}^{*}\left(G\right)$
[1]	$21.628$	$51.44$	$330.56$	$105.94$	$163.424$	$4744$	$1832$
[2]	$33.556$	$80.45$	$524.12$	$165.94$	$259.034$	$7558$	$2918$
[3]	$45.484$	$109.46$	$717.68$	$225.91$	$354.648$	$\mathrm{10,372}$	$4004$
[4]	$57.412$	$138.47$	$911.24$	$285.88$	$450.26$	$\mathrm{13,186}$	$5090$
[5]	$69.34$	$167.48$	$1104.8$	$345.85$	$545.872$	$\mathrm{16,000}$	$6176$
[6]	$81.268$	$196.49$	$1298.36$	$405.82$	$641.484$	$\mathrm{18,814}$	$7262$
[7]	$93.196$	$225.5$	$1491.9$	$465.79$	$737.096$	$\mathrm{21,628}$	$8348$
[8]	$105.124$	$254.51$	$1685.4$	$525.76$	$832.708$	$\mathrm{24,442}$	$9434$
[9]	$117.052$	$283.52$	$1879.04$	$585.73$	$928.32$	$\mathrm{27,256}$	$\mathrm{10,520}$
[10]	$128.98$	$312.53$	$2027.6$	$645.7$	$1023.93$	$\mathrm{30,070}$	$\mathrm{11,606}$

**Figure 11:**
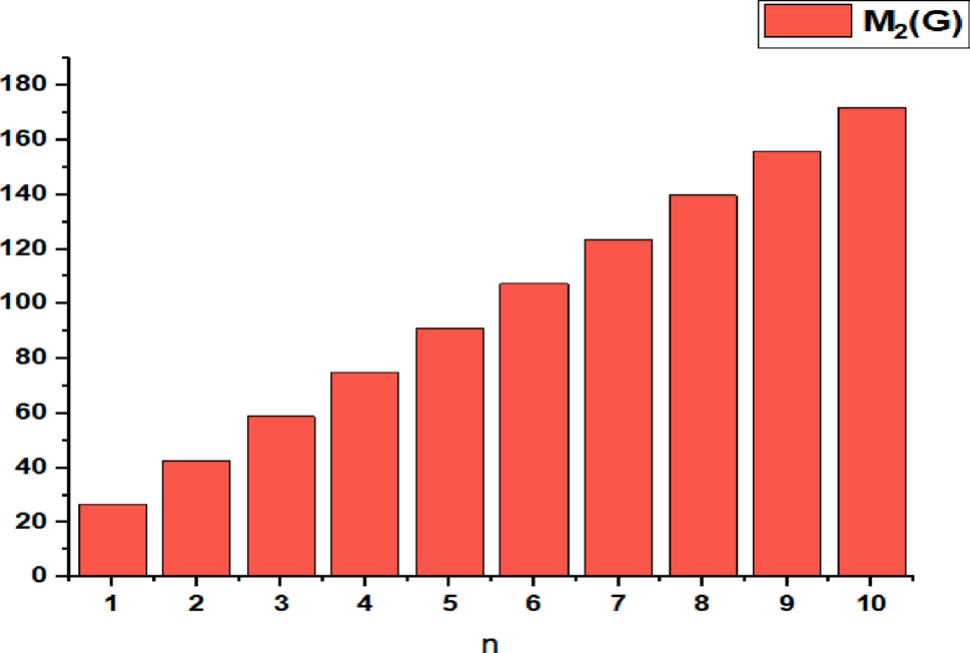
The comparison of $n$ and ${M}_{2}\left(G\right)$.

**Figure 12:**
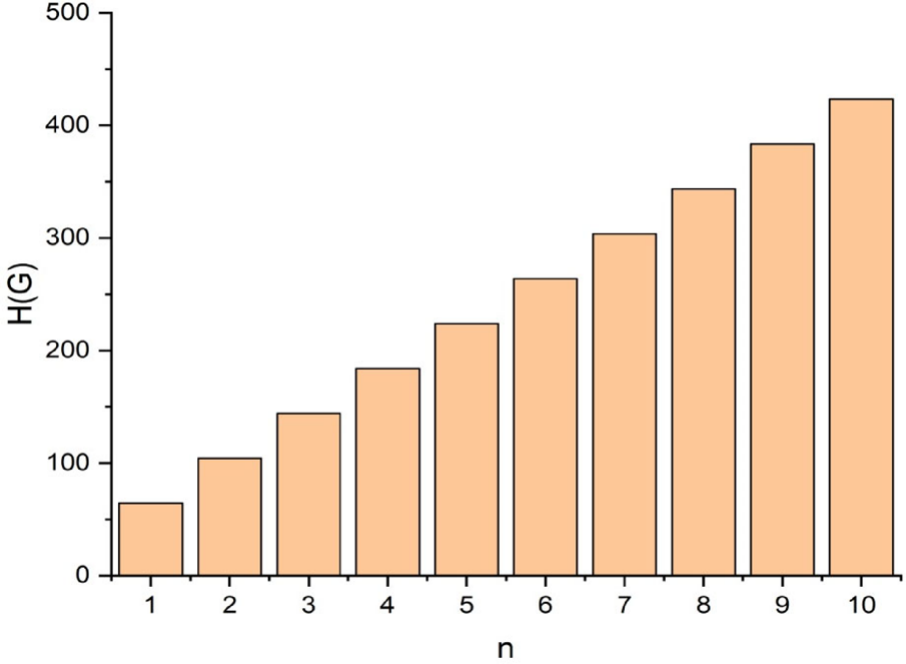
The comparison of $n$ and .$H\left(G\right)$

**Figure 13:**
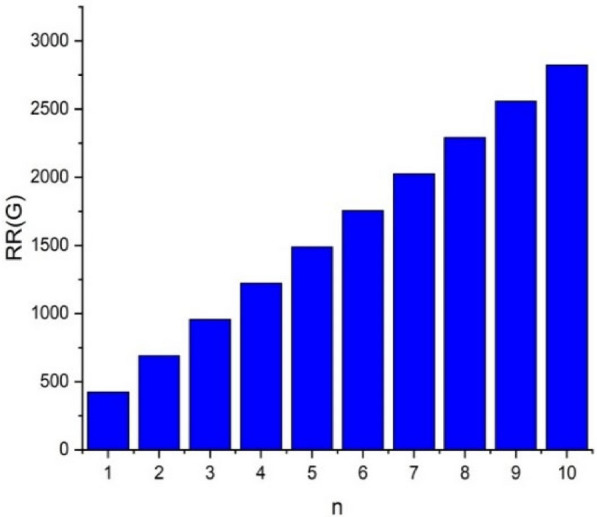
The comparison of $n$ and .$RR(\mathrm{G})$

**Figure 14:**
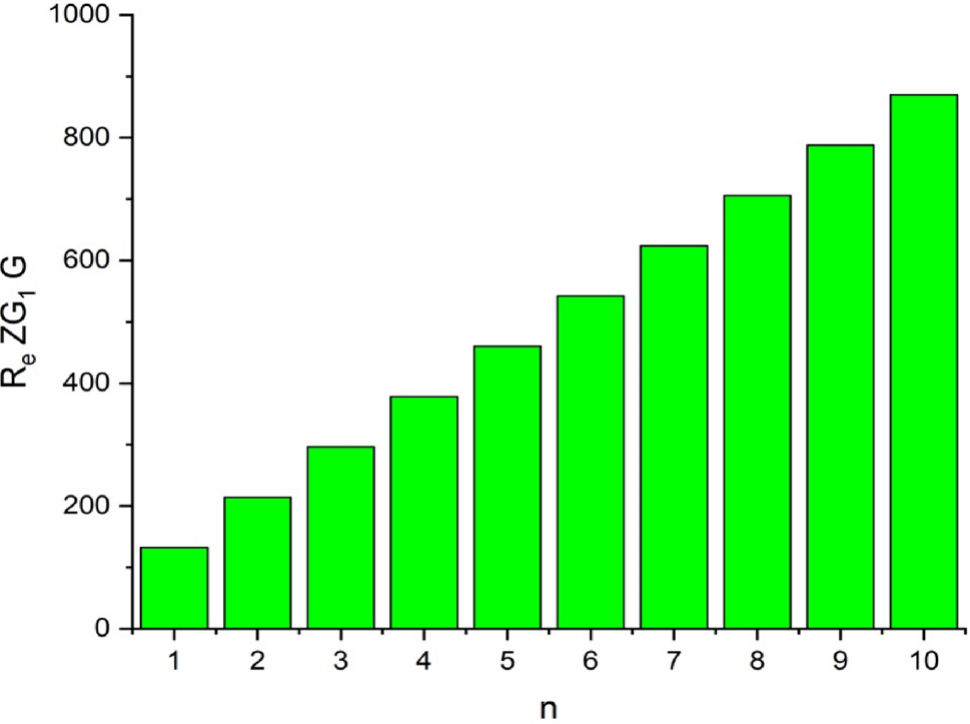
The comparison of $n$ and .$\boldsymbol{ }{\mathrm{R}}_{\mathrm{e}}{\mathrm{ZG}}_{1}(\mathrm{G})$

**Figure 15:**
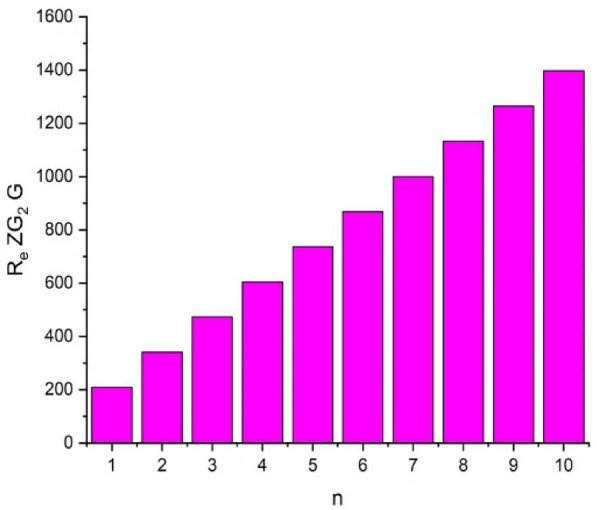
The comparison of $n$ and .$\boldsymbol{ }{\mathrm{R}}_{\mathrm{e}}{\mathrm{ZG}}_{2}(\mathrm{G})$

**Figure 16:**
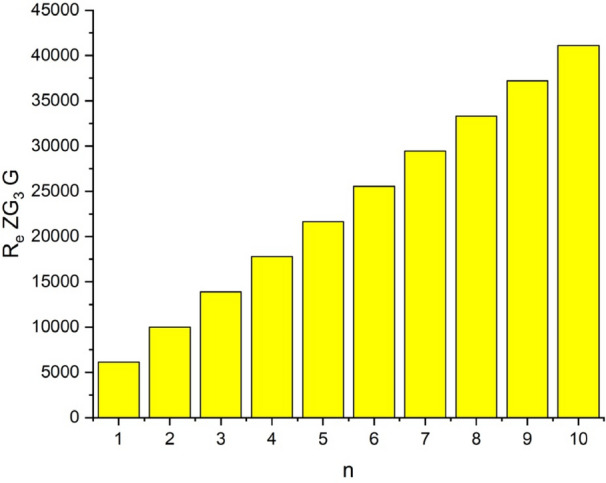
The comparison of $n$ and .$\boldsymbol{ }{\mathrm{R}}_{\mathrm{e}}{\mathrm{ZG}}_{3}(\mathrm{G})$

**Figure 17:**
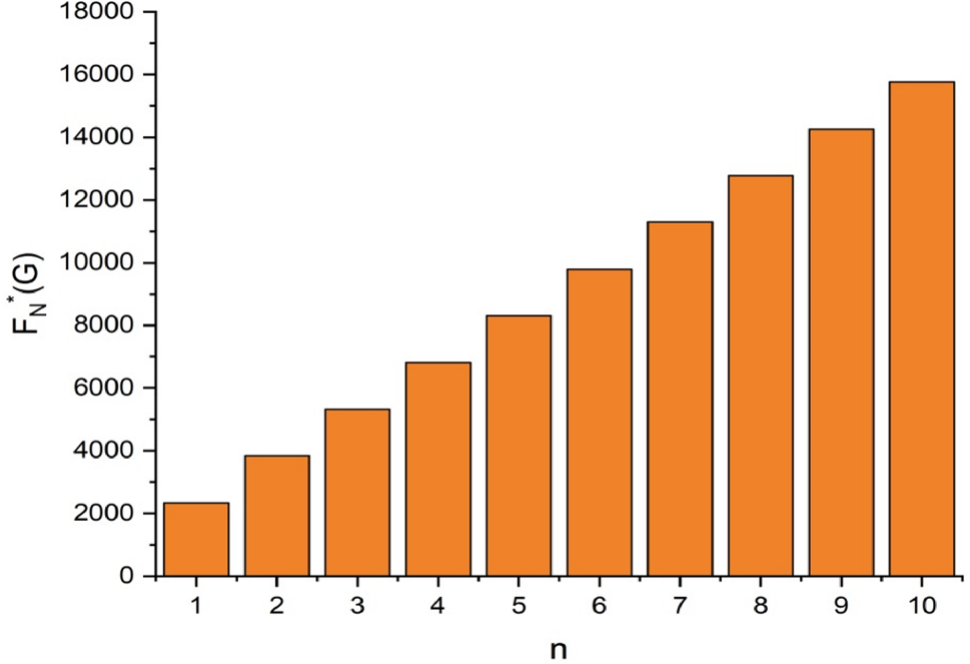
The comparison of $n$ and ${F}_{N}^{*}\left(G\right)$.

**Figure 18:**
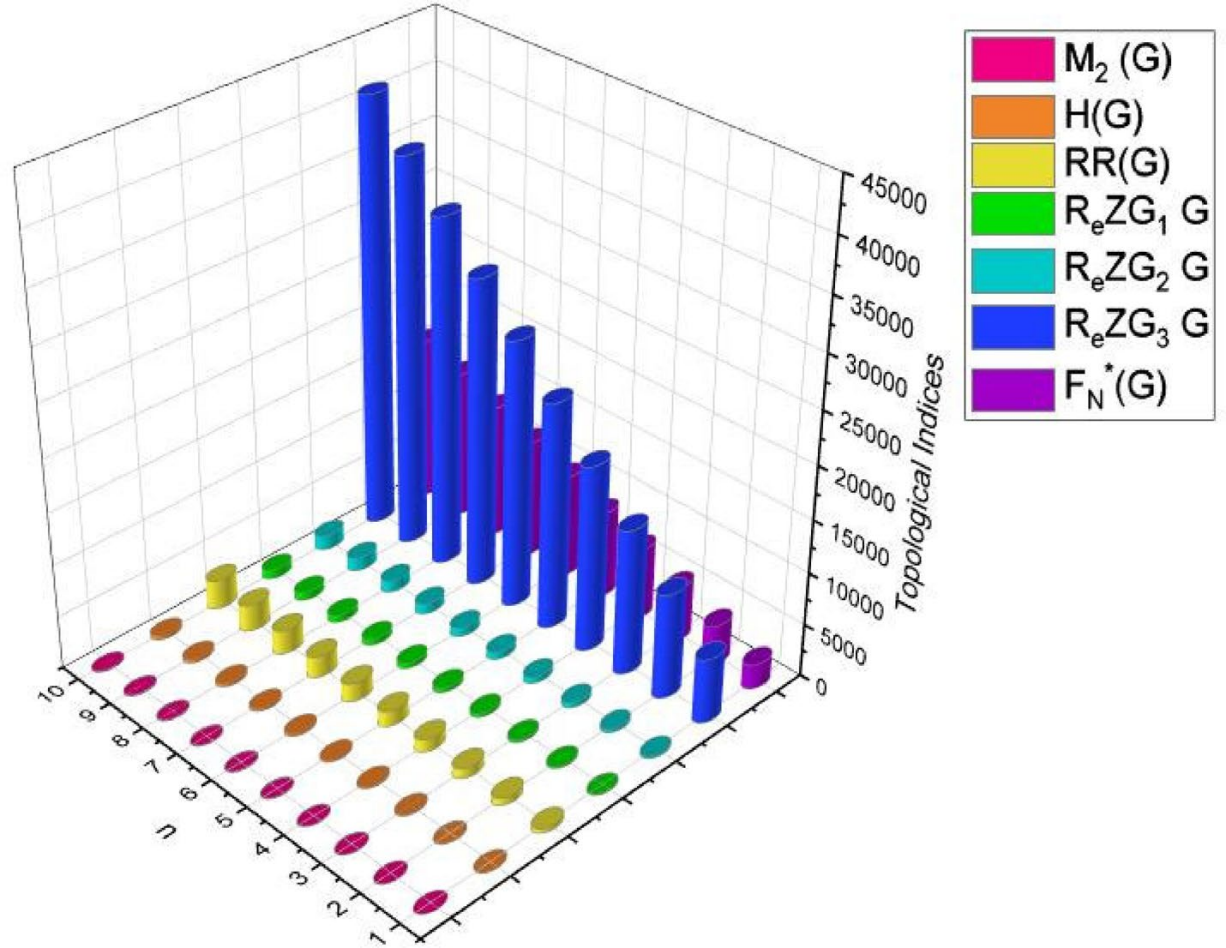
Comparison of topological indices for various values of $n$ in $ZNSL$.

From Figs. 11, 12, 13, 14, 15, 16, 17 and Table 4, it is easy to see a positive relationship between *n* and the considered topological indices. As we increase *n*, the topological indices also increase. Meanwhile, the comparative relationship of ${\mathrm{M}}_{2}\left(\mathrm{G}\right)$, $\mathrm{H}\left(\mathrm{G}\right)$, $\mathrm{RR}\left(\mathrm{G}\right)$, ${\mathrm{R}}_{\mathrm{e}}{\mathrm{ZG}}_{1}(\mathrm{G})$, ${\mathrm{R}}_{\mathrm{e}}{\mathrm{ZG}}_{2}(\mathrm{G})$, ${\mathrm{R}}_{\mathrm{e}}{\mathrm{ZG}}_{3}(\mathrm{G})$ and ${\mathrm{F}}_{\mathrm{N}}^{*}\left(\mathrm{G}\right)$ indices of $ZNSL$ for various values are presented in Fig. 18. Thus, Fig. 18 describe that all indices for $\mathrm{ZNOX}$ increase for increasing value of *n*. The increasing rate of ${R}_{e}Z{G}_{3}(G)$ is higher than that of other topological indices. This depicts that, ${R}_{e}Z{G}_{3}\left(G\right)$ achieved higher values than other classical Zagreb indices, which may have better correlation with the thirteen physicochemical characteristics of octane isomers.

**Table 4:** Comparison of ${\mathrm{M}}_{2}\left(\mathrm{G}\right)$, $\mathrm{H}\left(\mathrm{G}\right)$, $\mathrm{RR}\left(\mathrm{G}\right)$, ${\mathrm{R}}_{\mathrm{e}}{\mathrm{ZG}}_{1}\left(\mathrm{G}\right),$ ${\mathrm{R}}_{\mathrm{e}}{\mathrm{ZG}}_{2}(\mathrm{G})$, ${\mathrm{R}}_{\mathrm{e}}{\mathrm{ZG}}_{3}(\mathrm{G})$ and ${\mathrm{F}}_{\mathrm{N}}^{*}\left(\mathrm{G}\right)$.

[$n$]	${M}_{2}\left(G\right)$	$H\left(G\right)$	$RR\left(G\right)$	${R}_{e}Z{G}_{1}(G)$	${R}_{e}Z{G}_{2}(G)$	${R}_{e}Z{G}_{3}(G)$	${F}_{N}^{*}\left(G\right)$
[1]	$26.45$	$64.29$	$421.6$	$132.01$	$208.62$	$6120$	$2344$
[2]	$42.62$	$104.17$	$688.44$	$214.01$	$340.63$	$\mathrm{10,006}$	$3834$
[3]	$58.79$	$144.05$	$955.28$	$296.01$	$472.64$	$\mathrm{13,892}$	$5324$
[4]	$74.96$	$183.93$	$1222.12$	$378.01$	$604.65$	$\mathrm{17,778}$	$6814$
[5]	$91.13$	$223.81$	$1488.96$	$460.01$	$736.66$	$\mathrm{21,664}$	$8304$
[6]	$107.3$	$263.69$	$1755.8$	$542.01$	$868.67$	$\mathrm{25,550}$	$9794$
[7]	$123.47$	$303.57$	$2022.6$	$624.01$	$1000$	$\mathrm{29,436}$	$\mathrm{11,284}$
[8]	$139.64$	$343.45$	$2289.4$	$706.01$	$1132.6$	$\mathrm{33,322}$	$\mathrm{12,774}$
[9]	$155.81$	$383.33$	$2556.3$	$788.01$	$1264.7$	$\mathrm{37,208}$	$\mathrm{14,264}$
[10]	$171.98$	$423.21$	$2823.1$	$870.01$	$1396.71$	$\mathrm{41,094}$	$\mathrm{15,754}$

## Conclusion

In this research, we calculated various recently discovered molecular descriptors for two separate metal–organic networks. The molecular descriptors which we considered are ${M}_{2}\left(G\right)$, $H\left(G\right)$, $RR\left(G\right)$, ${F}_{N}^{*}(G)$, ${R}_{e}Z{G}_{1}(G)$, ${R}_{e}Z{G}_{2}(G)$ as well as ${R}_{e}Z{G}_{3}(G)$. The two metal–organic networks we considered are, Zinc oxide $\left(ZNOX\left(n\right)\right)$ and zinc silicate $\left(ZNSL\left(n\right)\right)$. The numerical and graphical comparative analysis of the considered molecular descriptors are also performed. The obtained results depict that ${R}_{e}Z{G}_{3}\left(G\right)$ achieved higher values than other classical Zagreb indices, which may have better correlation with the thirteen physicochemical characteristics of octane isomers. It is quite motivating to study the distance based topological indices for the Metal–Organic Networks. In the near future we will carry it out.

## Data Availability

All data generated or analysed during this study are included in this article.
